# The Validity of Using Analogue Patients in Practitioner–Patient Communication Research: Systematic Review and Meta-Analysis

**DOI:** 10.1007/s11606-012-2111-8

**Published:** 2012-06-15

**Authors:** Liesbeth M. van Vliet, Elsken van der Wall, Akke Albada, Peter M. M. Spreeuwenberg, William Verheul, Jozien M. Bensing

**Affiliations:** 1 NIVEL (Netherlands Institute for Health Services Research), PO box 1568, 3500 BN Utrecht, The Netherlands; 2 University Medical Center Utrecht, Utrecht University, Utrecht, The Netherlands; 3 Department of Clinical and Health Psychology, Utrecht University, Utrecht, The Netherlands

**Keywords:** communication, patient preferences, research design, doctor–patient relationships, systematic reviews

## Abstract

When studying the patient perspective on communication, some studies rely on analogue patients (patients and healthy subjects) who rate videotaped medical consultations while putting themselves in the shoes of the video-patient. To describe the rationales, methodology, and outcomes of studies using video-vignette designs in which videotaped medical consultations are watched and judged by analogue patients. Pubmed, Embase, Psychinfo and CINAHL databases were systematically searched up to February 2012. Data was extracted on: study characteristics and quality, design, rationales, internal and external validity, limitations and analogue patients’ perceptions of studied communication. A meta-analysis was conducted on the distribution of analogue patients’ evaluations of communication. Thirty-four studies were included, comprising both scripted and clinical studies, of average-to-superior quality. Studies provided unspecific, ethical as well as methodological rationales for conducting video-vignette studies with analogue patients. Scripted studies provided the most specific methodological rationales and tried the most to increase and test internal validity (e.g. by performing manipulation checks) and external validity (e.g. by determining identification with video-patient). Analogue patients’ perceptions of communication largely overlap with clinical patients’ perceptions. The meta-analysis revealed that analogue patients’ evaluations of practitioners’ communication are not subject to ceiling effects. Analogue patients’ evaluations of communication equaled clinical patients’ perceptions, while overcoming ceiling effects. This implies that analogue patients can be included as proxies for clinical patients in studies on communication, taken some described precautions into account. Insights from this review may ease decisions about including analogue patients in video-vignette studies, improve the quality of these studies and increase knowledge on communication from the patient perspective.

**Electronic supplementary material:**

The online version of this article (doi:10.1007/s11606-012-2111-8) contains supplementary material, which is available to authorized users.

## INTRODUCTIONS

Studies of the patient perspective on communication usually rely on clinical patients (CPs) who rate their practitioner’s communication.^1^,^2^ Other studies rely on analogue patients (APs)—patients and/or healthy subjects—who rate videotaped medical consultations while putting themselves in the shoes of the video-patient. These videotapes can be of real encounters (referred to as ‘clinical studies’) or scripted encounters (referred to as ‘scripted studies’). Scripted studies provide researchers the opportunity to vary and study specific elements of communication (e.g. compassionate remarks).^3^

Until now, insight into the reasons warranting the use of video-vignette studies with APs is lacking. Studies might use general, implicit rationales. Alternatively, there may be ethical considerations; not all communication can cautiousness be randomized in clinical care. Methodological advantages may be another reason. As stated above, scripted studies can investigate specific elements of communication. Additionally, CPs are often extremely satisfied with their practitioner^4^,^5^—perhaps because they feel dependent^6^ or because of social desirability^7^—leading to ceiling effects. It has to be established whether APs’ evaluations of communication can overcome ceiling effects, and which rationales underlie and strengthen the use of video-vignette studies.

While video-vignette studies are sometimes preferred over empirical studies, the former may have validity problems. With regard to internal validity in scripted studies, the question arises whether manipulations are successful, i.e. variations in empathy should be perceived as such. With regard to external validity, the question arises whether results are generalizable to CPs and clinical care, i.e. are APs able to adopt a video-patient’s perspective?

Considerable research has been conducted on how CPs perceive their doctor’s communication. CPs appreciate various types of affective communication: verbal empathy,^8^–^10^ social talk,^8^–^11^ non-verbal eye-contact^8^ and listening.^8^,^10^ Appreciated instrumental communication includes information-giving.^8^–^12^ Last, ‘patient-centeredness’ is an often studied ‘general’ communication style mostly associated with positive outcomes.^8^–^15^ Whether APs evaluate these communication elements similarly is largely unknown.

To summarize, we lack an understanding of the rationales for conducting video-vignette studies with APs; how both internal and external validity are increased and tested; how APs’ perceptions of communication correspond to CPs’ perceptions; and whether APs’ evaluations of communication overcome ceiling effects. An overview of these elements will provide more insight into when and how APs can be used in future studies. Therefore, a systematic review is conducted with the following research questions:

1. What are the rationales for conducting clinical and scripted video-vignette studies on medical communication with APs ?
2. What have video-vignette studies done to increase and test their internal and external validity?
3. How do APs perceive—affective, instrumental and general—communication elements?
4. Do APs’ evaluations of communication overcome ceiling effects?

## METHODS

### Identification of Studies

Pubmed, Embase, Psycinfo and CINAHL were searched in February 2012. Searches were not restricted to any parameter and focused on two central concepts: ‘analogue patients’ and ‘video’ (see the Online Appendix Supplementary data for search strategies used).

Studies were eligible for inclusion if they were about (verbal/nonverbal) communication between physicians/nurses and patients and: i) used video-vignette designs; ii) included APs (>18 years): healthy subjects, untrained or trained only for this study; patients not judging their own doctor/nurse; standardized patients viewing a videotaped consultation they took part in; and iii) used APs’ perceptions of physician’s/nurse’s communication as outcome measures (e.g., preferences, recall). Studies were excluded if: i) observers were trainers, research assistants, trained/experienced coders, examiners, medical students or faculty members; ii) APs’ comments did not include a quality judgment.

### Data

The following data were extracted from each study and summarized in Table 1: study characteristics and quality, design, rationales for conducting video-vignette studies with APs, attempts to increase and test internal and external validity, limitations, and APs’ perceptions of the studied communication elements.

**Table 1:** Characteristics and Results of Included Video-Vignette Studies

Author, year, country, quality	Aim of the study	Sample	Design	Method	Rationale for design & method	Internal validity	External validity	Results	Limitations
Aruguete & Roberts, 2000, USA, superior	To examine the effectiveness of affiliative and controlling communication styles for male and female physicians.	n = 146 undergraduate psychology students, 91 female, age range: 17–51 years, M=21	Cross-sectional; experimental study structured survey	APs viewed one out of four 5-minute videos in which a physician discussed a diagnosis of a peptic ulcer. This could be done in an affiliative or controlling communication style, either by a female or male physician. Post video measures: satisfaction, trust, motivation to comply, likelihood of recommendation, willingness to self-disclosure, recall.	An analogue design is used to examine the causal effects of variables (gender and communication style).	APs perceived the controlling and affiliative physicians as such.	The affiliative style was seen as more believable than the controlling style. Subsequent analyses were controlled for believability. APs were waiting for a medical consultation.	An affiliative style led to higher APs’ satisfaction, trust, self disclosure and motivation to comply, independent of physician gender. For male students, recall was lower for the affiliative style.	The behavior or APs might not reflect CPs’ behavior. APs were students and thus not representative of the patient population. Likelihood of disclosure etc was measured instead of real behavior.
Aruguete & Roberts, 2002, USA, superior	To determine the effect of physician race (white vs. black) and nonverbal behavior (concerned vs. distant).	n = 116 lay people, 84 % black, age M=23	Cross-sectional; experimental study structured survey	APs viewed one out of four 7-minute videos of a physician-patient interaction. The race of the physician and nonverbal concernedness were systematically varied. Post video measures: satisfaction, trust, motivation to comply, likelihood of recommendation, willingness to self-disclosure, recall.	The analogue design increases internal validity.	APs could distinguish between a nonverbal distance and concerned communication style.	APs were waiting for a medical consultation. To increase identification with the video-patient the camera depicted the physician while patient appearance and dialogue was minimal. The four videos were seen as believable.	Concerned nonverbal behavior led to higher APs’ self disclosure, motivation to comply, likelihood of recommendation, trust, satisfaction and recall, independent of physician race. For non-verbal distant physicians, men were more satisfied and the likelihood of recommendation was higher for same-race physicians.	The analogue design increases internal validity at the cost of external validity; whether APs perceptions compare to CPs’ perceptions is unsure. APs were young, Afro-American and in good health, thus not representative. Last, likelihood of disclosure etc was measured instead of real behavior.
Blanch et al., 2009, USA, superior	To determine the effect of medical students’ expressions of uncertainty (EOU) in a standardized visit.	n = 244 undergraduate students	Cross sectional: non-experimental study with standardized patients (SPs); structured survey	APs watched one out of 72 videos. Post video measures: perceived confidence, competence, compassion, communication, satisfaction and overall performance.	APs have been used to understand patient perceptions when the actual patient population is not available.	Levels of EOU were objectively coded by trained raters (only low scored were included).	None	EOU were related to lower scores of confidence, competence, satisfaction, liking and communication according to APs.	It is not certain whether the same results would be found with real physicians instead of students.
Blanch-Hartigan et al., 2010, USA, superior	To determine whether there is a gender bias in patient perceptions of patient-centered (PC) behaviors.	N = 384 undergraduate students, 265 female, 76 % Caucasian.	Cross sectional: non-experimental study with SPs; structured survey	Pre video measure: preference for patient-centeredness (PC) (PPOS). Next, APs received a neutral message or a message stressing the importance of either technical competence or PC. Then APs viewed one out of 61 videos. Post video measures: perceived compassion and competence. PPOS.	This methodology has been used in previous studies focused on patient perceptions when the actual patient population is not available. Moreover, this design controls for patient behavior and other factors that might influence results.	The medical students’ PC was objectively coded by applying the Four Habits Coding Scheme and RIAS.	None	AP judged a more PCphysician as more compassionate (following each message). A more PC male physician was seen as more competent following each message. A more PC female physician was seen as less competent after the neutral + technical message and more competent after the PC message.	APs were young and may be unrepresentative for the general population. APs had no interaction with the medical students. Research in clinical settings is needed.
Bradley et al., 2001, Australia, superior	To determine the effect of consultative versus authoritative communication on APs’ perceptions. Moderating effects of age and gender are determined.	N = 492 lay people, 333 female, age range: 17–84, M=39.5	Cross-sectional; experimental study structured survey	APs viewed in groups one 3-minute videotaped scenario in which the communication style of the physician was either consultative or authoritative. Post video measures: satisfaction, likelihood to comply with medial recommendations (adherence) and recall.	An experimental design has the advantage that ratings are made independently of confounding variables (e.g. previous exposures). Found results between female and male physicians can be attributed to gender instead of gender-based patterns of behavior.	Scripts were created with help of focus groups and a medical practitioner. Written scripts were validated by students and lay people/health professionals. APs could distinguish between a consultative and authoritative communication style.	Simulated situations evoke the same reactions as actual settings. APs read an introduction to the scenario to increase identification. The videos were seen as realistic (especially the authoritative style). APs could easily adopt to the patient’s role.	A consultative communication style increased APs’ satisfaction, but not adherence or recall. These latter outcomes were moderated by gender and age, i.e. a female consultative style increased adherence the most, while for young APs recall was highest with an authoritative male or consultative female.	More research is needed to replicate the findings in other conditions, other research methods and on more age categories.
Cousin & Schmid Mast, 2011, Switzerland, superior	To determine whether correspondence in non-verbal affiliative behavior between physician and patient is related to positive patient outcomes.	n = 58 students, 58 % female	Cross sectional; non-experimental study with CPs; structured survey	APs watched from 8 unscripted videos (4 including female doctors, 4 including male doctors) 2-minute silent excerpts and indicated their satisfaction, trust, competence and determined adherence. APs’ agreeableness was measured once.	The use of APs is not rare in the field of physician-patient communication. By standardizing the physician (i.e. all APs viewed all physicians), all variances in the dependent variables can be attributed to the APs level.	The physician’s affiliative behavior was objectively coded.	None	Higher agreeable APs react to the affiliative physician with higher competence, trust and adherence scores, but not with higher satisfaction scores. The higher competence scores moderated the influence on trust and adherence. Overall, high affiliativeness was related to better outcomes.	The generalizability to real patients is uncertain; APs had no real physical complaints and related distress and they were young.
Dowsett et al., 2000, Australia, superior	To explore preferences for and satisfaction with PC and doctor-centered (DC) communication in a breast cancer consultation (with the segments: diagnosis, treatment, prognosis).	n = 113 women treated for breast cancer and 48 of their relatives/friends	Cross-sectional; experimental study structured survey	APs watched 6 videos with either a good or bad prognosis. The doctor’s communication was systematically varied. After every style in every segment satisfaction was measured and after both styles per segment the preferred doctor was indicated.	Video-vignette studies produce less skewed satisfaction scores; APs can compare and contract different styles directly.	Oncologists and patients were involved in creating scripts. APs rated all doctor styles in every segment as ‘worst to best doctor available’; indicating they could discriminate between the two styles.	Both healthy participants and cancer survivors were included.	Perceptions of survivors and relatives/friends overlapped. APs preferred a PC approach. Predictors were: watching a poor prognosis and having a professional occupation.	Whether the results are generalizable to other participants (e.g lower SES, other cancer types) is uncertain.
Floyd et al., 1999, USA, superior	To determine the comfort levels with different communication approaches for conducting a HIV risk assessment.	n = 75 lay people: students, people from a senior center. 75 % female. Age range: 20–75 years.	Mixed method; experimental study qualitative; structured survey	APs viewed 10 videos of a HIV risk assessment interview, comprised of 5 areas. In every area, 2 different communication styles were created. After every style APs indicated their level of comfort.	None	Videotapes, aimed to display a variety of interview approaches, were evaluated by an independent expert panel.	None	A PC approach and close-ended interviewing techniques were most comforting.	Whether the results are generalizable to CPs is uncertain. Other factors than the varied communication may influence comfort.
Fogarty et al., 1999, USA, superior	To assess the effect of compassion in a breast cancer consultation, in which palliative treatment options are being discussed.	n= 123 breast cancer survivors, 87 healthy women.	Cross-sectional; experimental study; structured survey	APs viewed either a ‘standard’ video or a video with enhanced compassion. Post video measures: physician compassion, anxiety, recall, treatment decision, perceptions of physician, treatment choice.	None	The scripts were created based on audiotapes of real consultations.	Both healthy participants and cancer survivors were included. Four pilot focus groups were held. Videotapes seemed realistic and appropriate for the intended audience.	Perceptions of survivors and healthy women overlapped. The compassionate physician seen as: more compassionate, warm, sensitive, pleasant, higher on specific attributes. Anxiety and recall decreased.	Whether the results are generalizable to women with breast cancer (at the time they are making treatment decisions) is unknown.
Gask et al., 1989, UK, average	To examine the effect of an 8-week training for psychiatry trainees on the use of specific communication skills.	n = not reported, SPs	Cross sectional: non-experimental study with SPs; structured survey	Pre- and post- training, trainees had a consultation with a SP. The SPs watched the consultation on video, rated the trainees’ communication skills.	None	None	None	APs judged the trainees after the consultation better in explaining the link between somatic complaints and psychological distress.	None
Gerbert et al., 2003, USA, superior	To determine what kind of physician (race and ethnicity) is preferred.	n = 359 lay people. 61 % female.	Cross-sectional: experimental study: structured survey	APs viewed 6 doctors varying in gender and ethnicity. They indicated their favorite doctor after an introduction segment and after a preventive message and rated the doctors on different qualities (e.g. professional)	Through video-method a verisimilar experience is created, in which i) the constraints of availability and access of real life are overcome, ii) variables such as doctor’s age may be held constant.	None	None	Same-race and female doctors were preferred initially. After the preventive message APs preferred the PC female doctors even more. The preference for same-race doctors decreased.	None
Gilbert, 1998, 2004, USA, superior	To investigate which relational themes are communication by nurses’ (verbal/nonverbal) listening behavior during brief interactions and whether these themes reflect positive patient-nurse relationships.	n = 126 female students. Age range: 17–44 years	Cross sectional: non-experimental study with SPs; structured survey	APs watched six 30-second segments of nurse-patient interactions. After every segment, they rated the extent to which the nurses communicated relational themes and indicated their overall satisfaction.	None	None	None	Participants were more satisfied when a nurse communicated ‘trust’, ‘affection’, ‘composure’, ‘little difference’, ‘little formality’. Nonverbal simultaneous coordination communicates positive patient-nurse relational information.	All other influencing variables were controlled. Only white college women were included. Still unsure: i. whether nurses react the same in the clinical setting, ii. whether CPs would perceive relational themes equally
Gillotti et al., 2002, USA, superior	To understand which communication moves are associated with perceived competence in the delivery of bad news (disclosing a HIV diagnosis to a female patient)	n = 527 undergraduate students. 53 % female. Age range: 20–53.	Cross sectional: non-experimental study with SPs; structured survey	APs watched 3 videos in which a medical student provided the news. Post video measures: empathy and general communication skills.	None	Trained coders objectively coded behavior and this was related to APs ratings.	None	During bad news conversation information-verifying, -giving and -seeking, and small talk was not valued.	The perceptions of APs may not overlap with real patients: APs may focus more on the giver of bad news instead of own anxieties and may be higher educated.
Hall et al., 2009, 2009, USA, average	To examine the relation between medical students’ nonverbal sensitivity / rapport and APs’ impressions during a SP visit.	n = 244 students in psychology courses.	Cross sectional: non-experimental study with SP; structured survey	APs viewed a consultation between a medical student and SP. Post video measures: liking, compassion, satisfaction, quality of communication, self-confidence.	APs are used when access to CPs’ impressions is impractical or impossible.	Rapport and interpersonal sensitivity were objectively measured.	None	More nonverbal sensitivity led to higher ratings of liking, compassion. APs’ ratings of satisfaction, competence, good communication, confidence and calm were positively related with observed rapport.	The generalizability of APs’ findings to CPs remains to be discovered.
Harrigan & Rosenthal, 1983, USA, superior	To determine the effect of physician’s nonverbal behavior.	n = 118 psychology students. 60 % female. Age ranged between 17–25.	Cross sectional: experimental study: structured survey	APs watched 24 silent video segments in which a physician’s trunk position, head nodding and open arm/leg postures were systematically varied. After every segment the behavior was judged on different ratings, measuring rapport.	None	None	Patients could only view the physician.	Physicians who leaned backward, with their arms uncrossed and nodded their head received higher rapport ratings.	None
Haskard et al., 2009, USA, superior	To determine the effect of verbal and nonverbal affective and instrumental communication of nurses and patients on their satisfaction with a consultation and each other. This was related to APs’ perceptions.	n = 4 female naive raters (trained)	Cross sectional: non-experimental design with CPs; structured survey	Patients and nurses judged the consultation. APs watched silent videos of these consultations. They rated different affective (sensitive/caring) and instrumental (professional and negative/rushed) communication aspects.	None	None	None	When APs judged nurses’ nonverbal communication as more caring/sensitive+ less negative/rushed, CPs were more satisfied with the nurse. When APs judge nurses’ nonverbal communication as less negative/rushed, nurses were more satisfied with the consultation.	None
Johnson et al., 1988, USA, average	To determine the effect of physician’s expressed uncertainty when prescribing antibiotics on satisfaction.	n = 80 lay people	Cross sectional: experimental study: structured survey	APs watched one out of five videos. Video 1+2: no expression of uncertainty Video 3: uncertainty was expressed but ignored. Video 4+5: uncertainty was expressed and the doctor consulted a textbook (4) or computer (5). Post video measures: satisfaction.	By using videotapes, one aspect of the patient-physician interaction can be isolated and manipulated, while the remainder of the encounter is held constant.	APs’ ratings of perceived uncertainty were indeed higher when physicians expressed uncertainty.	APs were asked the question in the ‘I’ form. APs were waiting for a medical consultation, so they were sensitized to judge communication.	When a physician expressed more uncertainty, APs’ satisfaction ratings decreased.	The study was not conducted during a genuine medical encounter, which can limit the generalizability of the findings to real patients.
Kaaya et al., 1992, UK, average	To examine the effect of an 8-week training for psychiatry trainees on the use of specific communication skills.	n = not reported, SPs	Cross sectional: non-experimental study with SPs; structured survey	Before and after the training, trainees conducted a consultation with a SP. The SPs watched the consultation on video and rated the trainees’ communication skills.	None	None	None	APs judged the trainees post-consultation better in: i) explaining the findings from physical examination, ii) explaining the link between somatic complaints and psychological distress.	None
Koss et al., 1997, USA, average	To explore the effect of nonverbal behavior (positivity) on CPs’ satisfaction. This was related to APs’ perceptions.	n = 12 students	Cross sectional: non-experimental study with CPs; structured survey	Consultations between patients and doctors were videotaped. CPs gave their satisfaction ratings post-consultation. APs rated 20-seconds silent video excerpts on level of positivity.	None	None	None	APs’ ratings of positivity were higher for female doctors. There was no relationship with CPs’ satisfaction.	None
Mazzi et al 2011, Italy/UK/Netherlands/Belgium, superior	To study the quality of physician’s responses to patients’ negative emotions (i.e. cue/concern) in an OSCE setting.	n = 259	Cross sectional: non-experimental study with SPs; structured survey	APs viewed 4 videos of medical interactions and provided for each video an overall quality judgement. Next, for every video, 4 fragments of a patients’ expression of a cue or concern and the medical student’s reaction hereon were shown. AP judged the appropriateness of every reaction.	All APs viewed all videos, while physician’s reactions were coded, increasing standardization of quality assessments. This approach enabled the investigation of APs’ background characteristics on judgements. Next, the use of APs is a widely used methodology.	The cues/concerns and reactions were coded using VR-CoDES.	None	APs appreciated reactions which provided space the most. Especially explicit empathic reactions were appreciated. Next, individual quality assessments were influenced by the general impression from the consultation. Last, younger, higher educated APs were most critical.	APs were not emotionally engaged in the consultation and only from Western-European countries. Moreover, the validity of APs assessments needs to be determined.
Mazor et al., 2005, USA, average	To assess the correspondence between OSCE checklist scores and APs’ perceptions of communication, and whether specific behavior is related to APs’ satisfaction.	n = 111 lay people. 27 % female.	Cross sectional: non-experimental study with SPs: structured survey	APs watched five videotaped consultations. Post video measures: satisfaction with the doctor’s communication Next, the importance of the different checklist items was assessed.	It is important to have more raters, to increase reliability of scores.	None	None	APs were more satisfied when the physician: i) presented information clearly, ii) moved through the encounter efficiently. The OSCE checklist scores did not correspond to APs’ perceptions of communication.	None.
Mazor et al., 2007, USA, average	To investigate the processes raters (APs, SPs, doctors) use when judging professionalism of medical students, in an OSCE setting.	n = 3 lay people	Qualitative: non-experimental study with SPs; with think aloud technique	All raters watched 20 videos and expressed all their thoughts about professionalism on the following domains: introduction, respect, verbal and nonverbal communication, physical examination, overall conduct.	None	None	None	There was much variation between and within raters on how behavior is evaluated. APs appreciated information-giving.	None
McKinstry, 2002, UK, superior	To determine whether patients prefer a shared or directed approach in the decision making process of general practice consultations.	n = 410 lay people	Cross sectional experimental study; structured interviews/survey	APs watched in groups one out of five scenarios, both styles were viewed. Post video questions: i. which doctor do you prefer, ii. which doctor seems like your own doctor, ii. what is the difference between the two doctors?	None	For every scenario, the two versions were played by two different actor pairs. The 20 videos were shown to APs, revealing that the two styles differed on power, authority, length of time etc.	APs were waiting for a medical consultation.	For medical problems, a directed approach was valued, but not for depression and lifestyle. APs preferring a shared style were younger, of a higher social class, smokers.	None
Mumford et al.,1987, USA, average	To determine whether communication improves after a psychiatry clerkship.	n = 5 naive raters (trained)	Cross sectional: non-experimental study with SPs; structured survey	Medical students conducted a consultation with a SP pre and post a psychiatry clerkship. Videos were rated by APs on process communication.	Consultations with SPs were chosen because real patients vary, influencing students’ communication. Real consultations were also more difficult to plan.	None	APs’ ratings were compared with different measures, e.g. how much students were satisfied with their own work.	According to APs, students improved in the process area of communication.	None
Quilligan & Silverman, 2012, UK, superior	To study the effect of (different types of) summary in doctor–patient interactions	n = 2 SPs	Case study: non-experimental study with SPs; qualitative analysis	A videotaped interaction between a medical student and SP was analysed for summarizing. Then the student and SP viewed the video, which was stopped at summaries, and commented on the purpose and effect of the summary. These comments were qualitatively analysed.	None	None	None	According to SPs, the use of summary increased accuracy and let them know they had been heard. However, consistent incorrect summaries made them feel they were not listened to and made them question whether they have been clear.	SPs played a role, which may not reflect real situations. SPs may be more assertive and have more knowledge about summarizing. Research in clinical care is warranted.
Quirk et al., 2008, USA, superior	To define ‘caring’ from a patient perspective.	n = 46 lay people	Qualitative: non-experimental with SPs; focus groups	APs watched two videos; one with high caring and another one with low caring behavior of a doctor. They discussed the behavior in focus groups.	None	None	None	APs perceived different physicians’ themes as caring: i) communicate effectively, ii) arrange to meet healthcare needs, iii) respectful, iv) empathic. Within these themes, specific behaviors perceived as caring varied.	None
Roberts & Aruguete, 2000, USA, superior	To determine the effect of task- and socioemotional behavior of doctors when discussing a diabetes diagnosis.	n = 93 lay people. 44 % female.	Cross sectional: experimental study structured survey	APs watched one out of four videos in which the doctor’s behavior was high or low on socioemotional and task behavior. Post video measures: trust, recommendation, satisfaction and recall.	With experimental studies, the isolated effect of physician behavior on patient responses can be studied.	47 APs scored each videotape on socioemotional and task attributes of the doctor. They could recognize both behaviors, although socio-emotional better.	APs only saw the face of the doctor, as if he was directly talking to them. 47 participants also rated the believability of the interaction. The videos were seen as believable. APs were waiting for a medical consultation.	Socio-emotional behaviors led to higher levels of: satisfaction, recommendation, trust and words recalled. Task behavior did not influence outcomes.	The reactions of APs may not reflect how patients would react in actual consultations.
Roter et al., 2008, USA, superior	To assess the effect of nonverbal sensitivity on APs’ satisfaction and knowledge after watching a standardized genetic counselling consultation.	n = 559 lay people	Cross sectional: non-experimental study with SPs; structured survey.	Genetic counsellors (GCs) conducted a consultation with a SP. APs watched a video on prenatal screening or breast cancer. Post video measures: knowledge and satisfaction scores.	None	None	The following inclusion criteria for APs applied: Prenatal: under age of 35, having had a pregnancy (or partner had pregnancy). Cancer: Over 18 years of age, a family history of cancer.	GCs’ nonverbal sensitivity increased APs’ knowledge scores. GCs’ nonverbal sensitivity decreased satisfaction scores.	The attitude of APs might be differed from those of CPs; they were all college-educated.
Saha & Beach, 2011, USA, superior	To test the influence of a PC communication style on APs’ evaluation of the physician and acceptance of clinical recommendations.	n = 48 subjects over age 40 with a coronary artery disease (CAD) or risk of CAD	Cross-sectional; experimental study; structured survey	AP viewed one video, with either a high or low PC physician. Post video measures: competence, trust, liking, comfortable and overall evaluation. Next, the necessity of treatment was determined and likelihood to undergo treatment.	By using this design, the effect of specific variables could be studied, independent of other factors such as patient context. Next, while judging an unknown doctor high ceiling effects of CPs’ evaluative ratings could be overcome.	Patients, physicians and researchers helped to create the scripts and judged the final scripts on content.	To increase APs empathic involvement, the camera depicted the physician and either a female or male patient voice was used. Moreover, a brief introduction about the patient was given before seeing the video. Only participants at risk of/with CAD were included. APs were recruited at the care clinic. Patients, physicians and researchers found the final scripts realistic.	A PC style was seen as more competent, trustworthy, increased feelings of comfort and global evaluations, mainly by higher educated APs. Next, following a PC style APs felt a higher necessity for treatment and intention to undergo treatment, with less need for a second opinion.	In real interactions, evaluations and decision making are influenced by many variables not taken into account in this study.
Shapiro et al., 1992, USA, superior	To determine the effect of communication style when presenting ambiguous mammography results.	n = 40 healthy women	Cross sectional: experimental study: structured survey	APs watched the presentation of mammography results in either a worried or non-worried way. Pre video measure: anxiety. Post video measures: anxiety, recall, perceived severity.	The APs methodology is used when interventions in real consultations are impractical and impossible. Ethical constraints are important in this study.	Experts were involved in creating the scripts. A pilot study with 22 women was done; the videos differed on level of worriedness.	Participants had to have a high risk of cancer themselves. A brief introduction about the patient was given before seeing the video. APs could identify with the video-patient and the videos were perceived as realistic in the pilot study.	The worried presentation of information decreased APs’ recall, increased anxiety and the perceived severity of the situation.	The absence of interaction between the doctor and patient (as APs only viewed videos) hampers the generazability to real interactions.
Schmid Mast et al., 2005, Switzerland, superior	To determine the effects of different communication styles when providing a breast cancer diagnosis.	n = 159 major students	Cross sectional: experimental study; structured survey	APs watched a video in which a doctor used a PC DC or emotion-centered (EC) style. Pre video measures: mood. Post video measures: mood, satisfaction and perceptions of communication.	No cancer patients were included as APs because: i) it is not ethical to let them watch videos of a cancer consultation, ii) they may reflect on their own memory instead of communication in the video.	The three conditions were RIAS coded and indeed differed on communication.	APs were asked how well they could identify with the video-patient; no difference were observed between the 3 conditions.	APs were more satisfied with a PC approach and evaluated this doctor better. A PC approach led to lower increases in tension/depression.	The generalizability to real patients is uncertain. Only young, high educated women were included, while the average breast cancer patient is older.
Schmid Mast et al., 2008, 2011, Switzerland, superior	To determine the effect of various nonverbal and verbal behaviors on APs’ impressions.	n = 163 major students. 63 % female.	Cross sectional; non-experimental study with CPs; structured survey	APs watched from 11 unscripted videos 2-minute excerpts and indicated their satisfaction and perceived dominance (for the analysis on dominance, 8 videos were used; 4 including female doctors, 4 including male doctors)	The APs approach has been effectively applied in several studies to obtain representative measures. Using this methodology has the advantage of standardizing the physician.	None	APs are potential patients.	Concerning non-verbal behavior: APs were more satisfied with female doctors and when a physician displayed gender congruent behavior. Concerning (non)verbal behavior: APs perceived dominant behavior as more dominant for females than for male doctors and interpreted this more negative for females.	APs were young and high educated, which may hamper generalizability.
Swenson et al., 2004, 2006, USA, superior	To investigate preferences for a PC or DC approach when discussing complementary and alternative medicine (CAM) use	N = 250 lay people.	Mixed method; experimental study and qualitative; structured survey	APs watched a scenario in which CAM use was discussed, in a PC and DC style. Both styles were scored on: satisfaction and characteristics. For qualitative analysis the following two questions were asked: i) which of the doctors did you prefer, and why, ii) what were the differences between the two doctors?	By including APs less skewed distributions may be found. Using this methodology, all other variables can be controlled.	Expert panels helped to create the different scripts. The videos were shown to interns, which determined face validity.	In video research the same variations in preferences are found as in clinical studies with CPs. APs were waiting for a medical consultation.	A PC approach was valued, mostly by: younger, higher educated APs who were CAM users and had a PC physician. Qualitative analysis showed that APs’ evaluated the same behavior in different ways.	It is uncertain whether responses to videos are similar to responses of patients in the real clinical setting.
Willson & Mcnamara, 1982, USA, superior	To investigate how different levels of courtesy and competence influence APs’ perceptions with care.	n = 127 undergraduate students	Cross sectional: experimental study: Structured survey	APs watched one out of four videos in which a nurse discussed a sore throat with a patient. The videos varied on levels of competence and courtesy (high/low). Post video measures: satisfaction, intended compliance perceived courtesy and perceived competence.	Variations in quality of communication cannot be varied and studied in clinical care out of ethical constraints, so this approach offers a solution.	Clinical algorithms were used to create the technical parts of the script. In a pilot study, AP classified 80 % of communication fragments correctly. All APs also rated the courtesy/competence behavior, which were two different factors. Competence influenced level of courtesy but not vice versa.	In a pilot study APs thought the videos were credible. APs were given information on the medical condition, and were asked to think back of the last time they had this problem.	Courtesy led to higher satisfaction, but competence both to higher satisfaction and intended compliance.	Whether the results of APs’ viewing a videotape are the same as CPs’ reactions is unclear.

Quality of studies was assessed^16^ by applying the Research Appraisal Checklist (RAC).^17^ The RAC consists of 51 items covering the quality of title, abstract, introduction, methodology, data analysis, discussion, and style/form. Each item is scored on a 1–6 scale, so total scores can vary between 0 and 306 points with three quality categories: i) Below Average (0–103 points), ii) Average (103–204 points), iii) Superior (205–306 points).

### Meta-Analysis to Determine Ceiling Effects

To determine whether APs’ evaluations of communication (e.g. satisfaction, preferences) overcome ceiling effects, a random-effects multivariate meta-regression analysis^18^ was performed using the statistical package MLWIN 2.02.^19^ The following quantitative data was abstracted for each evaluation: M, SD, range. For each study the number of participants, videos viewed per participant and available videos was abstracted. For each evaluation, using various scales, the mean score was transformed to a 0–100 score^20^ using two formulas; for scales starting at 1: ((mean-1)/(range-1))x100, for scales starting at 0: ((mean/range))x100. Authors were contacted to provide relevant data not presented in the articles.

## RESULTS

**Figure 1.**
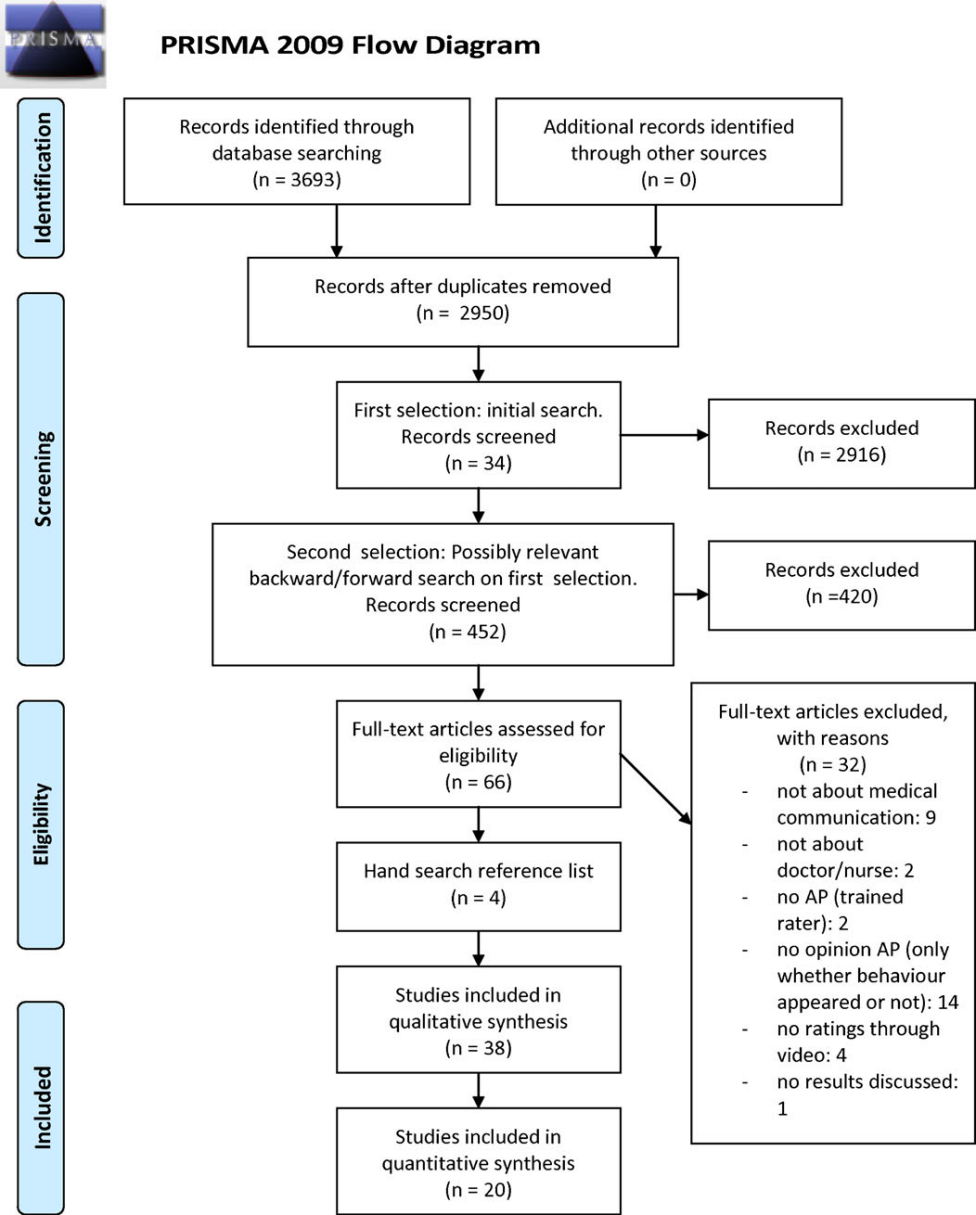
PRISMA flowchart of the inclusion procedure.

The 2950 references initially found were reviewed on title/abstract (and if necessary on full-text) to determine whether they: a) were about communication, b) used a video-vignette design, c) included APs. A random 10 % of the articles were independently checked on these criteria by two authors (LV and JB); interrater agreement exceeded 95 %. Thirty-four articles met these criteria and a forward- and backward reference search was performed. Four hundred and fifty-two new articles were reviewed in the aforementioned manner, resulting in 32 additional articles. These 66 articles were explored full-text on the final criteria: a) a focus on doctor/nurse-patient communication, b) inclusion of APs who viewed videos and judged the communication. Thirty-four articles met all criteria. Their references were hand-searched, resulting in four extra articles. Accordingly, 38 articles were included (see Fig. 1) that were based on 34 studies; some studies produced multiple articles.^21^–^28^

### Description of Included Studies

#### Study Characteristics and Quality

All studies were published in English, between 1982 and 2012, and conducted in the USA^3^,^21^–^26^,^29^–^48^ (n = 24), Switzerland^27^,^28^,^49^,^50^ (n = 3), UK^51^–^54^ (n = 4), Australia^55^,^56^ (n = 2), and an European setting.^57^ Studies were performed in general care 21–31,33–43,48,50,51,54,55,57 (n = 25), oncology^3^,^32^,^47^,^49^,^56^ (n = 5), psychiatry^46^,^52^,^53^ (n = 3), and genetic counseling.^45^

Most studies included lay people 21,22,29,31–36,39,42,45,46,51,55–57 (n = 16)—who were trained in two studies^36^,^46^—or non-medical students 23–28,30,34,37,38,41,43,44,48–50 (n = 13). Some studies included cancer survivors^3^,^47^ (n = 2) or patients with/at risk of coronary heart disease (CAD).^40^ In three studies standardized patients viewed videotaped consultations they had participated in.^52^–^54^

As determined with the RAC, 8 studies^25^,^26^,^31^,^35^,^37^,^39^,^46^,^52^,^53^ were of average quality, the remaining of superior quality. Four articles (10 %) were independently rated by two authors (LV and AA); the quality category was agreed on. Differences in quality assessment for specific items were resolved by consensus. The studies of average quality mostly lacked quality in the areas of methodology (e.g. design) and introduction (e.g. problem definition).

#### Study Design

Eighteen studies were clinical.^23^–^28^,^30^–^32^,^36^,^37^,^39^,^41^,^44^–^46^,^50^,^52^–^54^,^57^ These included videos with standardized 23–26,30–32,39,41,44–46,52–54,57 (n = 14) or clinical^27^,^28^,^36^,^37^,^50^ (n = 4) patients. Sixteen studies were scripted;^3^,^21^,^22^,^29^,^33^–^35^,^38^,^40^,^42^,^43^,^47^–^49^,^51^,^55^,^56^ APs watched one^3^,^29^,^35^,^40^,^42^,^43^,^47^–^49^,^55^ (n = 10) or multiple^21^,^22^,^33^,^34^,^38^,^51^,^56^ (n = 6) videos. Six studies^21^,^22^,^32^,^34^,^39^,^51^,^54^ had a (partially) qualitative approach. Physicians’ communication was most often assessed 3,21,22,25–35,37–44,46–57 (n = 31), but some studies included nurses^23^,^24^,^36^ (n = 2) or genetic counselors.^45^

### Rationales for Conducting Video-Vignette Studies with APs

Twenty-one studies reported general, ethical or methodological rationales for conducting video-vignette studies with APs.^21^,^22^,^25^–^31^,^33^,^35^,^40^–^43^,^46^–^50^,^54^–^57^ According to general rationales, APs are representative for CPs.^25^–^28^,^30^,^33^,^41^,^46^,^50^,^57^ Scripted studies pointed out the ethical constraints of standardizing (negative) communication in real consultations.^47^,^48^ When providing methodological rationales authors argued that; ceiling effects may be overcome;^21^,^22^,^40^,^56^ reliability increases with multiple raters;^31^ scripted studies increase internal validity^42^ and investigate communication systematically;^21^,^22^,^29^,^33^,^35^,^40^,^43^,^55^,^56^ clinical studies can standardize physicians^27^,^28^,^33^,^41^,^50^,^57^ and assess the influence of background characteristics.^50^,^57^ One study included healthy subjects because including patients would be unethical and patients may be distracted by their own memories.^49^

### Validity

#### Internal Validity

All but one^38^ of the scripted studies tried to achieve internal validity by ensuring that their manipulations were successful. APs^40^,^55^,^56^ and experts^21^,^22^,^40^,^47^,^55^,^56^ were involved in creating the scripts, or content from clinical interactions was used.^3^,^48^ Furthermore, APs^29^,^35^,^42^,^43^,^47^,^48^,^51^,^55^,^56^ or experts^21^,^22^,^34^ concluded that communication varied between videos (manipulation check), but only three studies provided useful numerical data.^35^,^47^,^48^ Other studies objectively coded the studied communication in their videos.^25^,^26^,^30^,^41^,^49^,^50^,^57^

#### External Validity

In attempt to ensure external validity several (oncological) scripted studies focused on APs’ identification with the video-patient. Three studies measured (and ensured) the level of identification.^47^,^49^,^55^ Other studies included subjects at risk of developing cancer^47^ or CAD,^40^ included CAD patients,^40^ or included both healthy participants and cancer survivors;^3^,^56^ their perceptions overlapped and were merged for analyses. Other (non-oncological) scripted studies tried to increase APs’ identification in various ways; by depicting only the physician;^29^,^38^,^40^,^42^ decreasing patient dialogue;^29^,^42^ introducing the patient (via text or video);^40^,^47^,^55^ asking participants to remember the time they visited the doctor with a similar health problem;^48^ using personalized questions;^35^ and recruiting participants waiting for a doctor’s appointment.^21^,^22^,^29^,^35^,^40^,^42^,^51^

Furthermore, scripted studies often focused on video credibility to ensure external validity; APs stated that the videos were credible,^3^,^29^,^42^,^43^,^47^,^48^,^55^ while only five studies provided numerical data.^29^,^42^,^47^,^48^,^55^ Indirect evidence for external validity was provided by clinical and scripted studies stating that: APs are potential patients;^27^,^28^ differences in APs’ preferences equal those of CPs;^21^,^22^ and simulated and clinical situations evoke equal reactions.^55^ Last, one clinical study^46^ assessed external validity, i.e. medical students who were appreciated by APs reported more satisfying interactions with CPs.

Twenty-three studies mentioned generalizability as limitation.^3^,^21^–^30^,^34^,^35^,^40^–^45^,^47^–^50^,^54^–^57^ It was often questioned whether APs’ reactions equal CPs’ reactions^3^,^21^–^26^,^29^,^34^,^35^,^42^–^45^,^49^,^50^,^54^,^57^ and whether findings were generalizable to real—interactive—consultations^23^,^24^,^30^,^40^–^43^,^47^,^54^ or other participants (e.g. demographic characteristics).^23^,^24^,^27^,^28^,^41^,^45^,^49^,^50^,^55^–^57^ Research with CPs in real consultations was often recommended.^23^–^26^,^30^,^34^,^41^,^44^,^48^,^49^,^54^,^55^

### Perceptions of Communication

APs’ perceptions of communication were studied. Patient-centeredness was preferred overall to doctor-centeredness,^21^,^22^,^33^,^34^,^40^,^41^,^49^,^56^ but not for acute physical problems.^51^ (Non)verbal affective communication was overall associated with positive effects (on trust,^29^,^43^ satisfaction,^29^,^43^,^48^,^55^,^57^ anxiety-reduction,^3^,^47^ intended self-disclosure^43^), but inconsistent results were found on intended compliance^43^,^48^,^55^ and recall.^3^,^29^,^47^,^55^ Social talk was appreciated in general care,^34^ but not during bad news conversations.^44^ Appreciated nonverbal behaviors included; rapport,^25^,^26^ listening,^23^,^24^ (non)verbal gender-congruent behavior,^27^,^28^ affiliativeness,^50^ an open body posture combined with nodding,^38^ concernedness,^42^ while the effect of nonverbal sensitivity was inconsistent.^26^,^45^ Two studies^36^,^37^ compared APs’ perceptions with videotaped patients’ satisfaction of nonverbal behavior; one study^36^ found a positive relation. Instrumental communication produced mixed results. In general care, information provision^31^,^39^,^54^ and little expression of uncertainty^30^,^35^ were appreciated, while the effect of competence was inconsistent.^29^,^48^ Conversely, during bad news consultations information-exchange was negatively evaluated.^44^

### Ceiling Effects

A random-effects multivariate meta-regression model compared the transformed means of 64 evaluations for 20 studies.^3^,^21^–^24^,^27^,^28^,^30^,^31^,^35^–^37^,^40^–^43^,^49^,^50^,^55^,^57^ The overall mean of APs’ evaluations was 54.28 on a 0–100 scale, 95 % CI: 47.99–60.57. (Single) mean evaluations varied between 24.00 and 82.00 while studies’ mean evaluations varied between 39.30 and 69.26, indicating also that no plateau effect occurred.

## DISCUSSION

This systematic review focused on the rationales, methodology and outcomes of medical video-vignette studies with APs. Scripted studies provided more specific rationales for using video-vignette designs with APs than clinical studies and directed more efforts at increasing/testing internal and external validity. APs’ perceptions of communication overlapped generally with CPs’ perceptions. Meanwhile, their evaluations overcame ceiling effects. These results have interesting methodological, theoretical and practical relevance.

Scripted studies paid the most attention to increasing the designs’ methodological soundness. Specific methodological rationales for conducting video-vignette studies with APs were provided, such as the opportunity to study communication systematically. This fills a gap in clinical care studies, in which only correlations, but no causality between communication and outcomes can be determined.^58^,^59^ Unfortunately, some scripted studies included container-concepts of communication (e.g., patient-centeredness). When positive effects are found, it remains unclear which specific element(s) of communication influenced outcomes.^15^,^58^ Additionally, as argued, when videos are watched by multiple APs, the reliability of assessments increases.^60^,^61^

Another argument for including APs was that their evaluations can overcome ceiling effects. APs’ evaluations were indeed not high; averagely 54.28 on a 0–100 scale. By comparison, a meta-analysis of CPs’ satisfaction ratings showed an average score of 80.00 (0–100 scale).^20^ Moreover, a recent study compared CPs’ satisfaction scores with those of APs viewing these videotaped consultations. Mean score (1–6 scale) for CPs was 5.8, while for APs it was 4.0 (p < 0.001).^62^ APs’ ratings thus seem to overcome this limitation of CPs’ evaluations.^4^,^5^ Accordingly, these and other methodological rationales provide strong foundations for conducting video-vignette studies with APs.

To achieve internal validity, APs reflected on manipulations in scripted consultations. Unexpectedly, ‘experts’ (doctors/researchers) were not often asked to comment on manipulations, although they may have insight into the manipulations’ (theoretical) success. Moreover, little information was provided on how exactly scripts were created, i.e. it often remained unclear what input researchers used to develop scripts and at what point(s) the scripts were validated.

Focusing on external validity, some studies argued that APs’ perceptions overlap with CPs’ perceptions. However, none of these studies determined whether APs watching videotaped consultations and CPs in these consultations overlapped on outcome measures. As stated earlier, such a study has recently been performed.^62^ In this study—taking into account CPs’ skewed satisfaction scores—APs’ and CPs’ evaluations were correlated. Additionally, a meta-analysis in psychology^63^ showed that lay people can make reliable judgments for (non)verbal communication based on brief (clinical and scripted) videotaped interactions.

Theoretical evidence supporting the external validity of APs can be found in simulation theory and is supported by neuro-cognitive studies on empathy. According to simulation theory, we infer other persons’ mental states by matching their states with resonant states of one’s own mental state.^64^ Neuro-cognitive studies show that the brain’s mirror neurons fire when a particular action is carried out or observed.^65^ They form the basis for empathy,^66^–^69^ as they are involved in experiencing and observing emotions in others^70^ and allow people to adopt another person’s perspective.^71^ Indeed, some oncological scripted studies included survivors alongside healthy participants. Their perceptions overlapped, indicating that healthy people can put themselves in the shoes of (cancer) patients.^72^

However, the methodological and theoretical rationales and advantages of using APs as proxies for CPs are relevant only when APs’ perceptions of communication are applicable in clinical practice, which is mainly supported by our results. APs’ perceptions of communication overlap mostly with those of CPs. A few—seemingly—contradictory findings were found. APs disliked information-exchange during bad news conversations, while CPs mostly valued this behavior. However, CPs often report receiving too much information during these conversations.^73^–^78^ Besides, while most studies point to the positive effects of patient-centeredness, a study with APs^51^ and review on CPs^12^ found that for purely physical complaints, a patient-centered style may be suboptimal.

Despite these promising results, various aspects should be taken into account when interpreting APs’ perceptions for clinical practice. First, in one study APs’ perceptions were unrelated to CPs’ satisfaction scores. The considerable age difference (students versus seniors) may be responsible for this finding, as age influences communication preferences.^79^–^81^ Future studies should take background characteristics influencing preferences—e.g. gender,^81^,^82^ education^83^,^84^—into account. Consequently, students should not be included as APs merely for convenience. Second, the diversity in APs’ evaluations should be kept in mind. The long-term doctor–patient relationship possibly influencing CPs’ evaluations cannot be captured by studies using APs. Thus, as video-vignette studies make it possible to disentangle the effect of various communication elements, these elements should afterwards be tested in clinical care.

### Limitations

This review has its limitations. First, the literature is inconsistent in the terms used for “analogue patients”. To include all relevant articles, both forward and backward reference searches on possible relevant articles were performed and included studies’ references were hand-searched. Future studies should use the term “analogue patients” consistently. Second, we excluded trained observers, but included lay people trained for this specific study. As studies may have used inconsistent labels, we screened for detailed information on observers. Despite these precautions taken, inadequately indexed and little cited relevant studies may have been missed, as we used a top-down search strategy.

## CONCLUSION AND FUTURE STUDIES

Scripted video-vignette studies increased their methodological soundness by providing specific rationales for conducting video-vignette studies with APs and increasing (internal and external) validity. In keeping with simulation theory and neuro-cognitive studies, APs’ perceptions of communication overlapped largely with CPs’ perceptions—while overcoming ceiling effects. However, it may be necessary to match participants on variables such as age and gender. Moreover, the effect of a long-term doctor–patient relationship on evaluations cannot be studied with APs. This leads to the conclusion—taking these precautions into account—that APs can provide knowledge on the patient perspective on communication.

Future—scripted—studies may benefit from the described elements to increase their methodological strength and provide more information about the process of ensuring validity. From this review we cannot conclude which communication elements—and outcome measures—can best be studied with APs. Ambady and Rosenthal^63^ suggested that communication with an affective component is fastest recognized because its evolutionary importance.^85^,^86^ Future studies could investigate differences between various types of APs. Research could build further on aforementioned work,^62^ comparing CPs’ perceptions with those from APs watching these consultations, taking into account differences in rating dispersion and focusing on background characteristics. This will raise the level of future studies in this promising research field, aimed at systematically unraveling the patient perspective on communication.

## Electronic supplementary material

Below is the link to the electronic supplementary material.

ESM 1 (DOCX 14 kb)
