# Supplementary material for: The Validity of Using Analogue Patients in Practitioner–Patient Communication Research: Systematic Review and Meta-Analysis
Source: J Gen Intern Med. 2012 Jun 15;27(11):1528–43. doi: 10.1007/s11606-012-2111-8 (PMC3475831; doi:10.1007/s11606-012-2111-8)
Supplement: Supplementary file 1 — (DOCX 14 kb) [file 11606_2012_2111_MOESM1_ESM.docx]

**Online Appendix – Search strategies used**

**Date of search: 17-2-2012**

**Pubmed:**

(("Patient Simulation"[Mesh] OR analogue patient* OR actor patient* OR simulated patient* OR placebo patient* OR standardized patient* OR lay person* OR healthy patient* OR healthy person* OR healthy subject* OR healthy participant* OR naïve patient* OR lay subject* OR lay people* OR naïve participant* OR naïve subject*) AND ("Video Recording"[Mesh] OR videotaped encounter OR video OR film OR audiovisual OR webcam)) OR analogue-patient*

**Embase (Excluding Medline. Exclude ‘map to preferred terminology):**

'analogue patient' OR 'analogue patients' OR 'lay people' OR 'actor patient' OR 'actor patients' OR 'simulated patient' OR 'simulated patients' OR 'placebo patient' OR 'placebo patients' OR 'standardized patient' OR 'standardized patients' OR 'lay person' OR 'lay persons' OR 'healthy patient' OR 'healthy patients' OR 'healthy person' OR 'healthy persons' OR 'healthy subject' OR 'healthy subjects' OR 'healthy participant' OR 'healthy participants' OR 'naive patient' OR 'naive patients' OR 'lay subject' OR 'lay subjects' OR 'naive participant' OR 'naive participants' OR 'naive subject' OR 'naive subjects' AND [embase]/lim AND videorecording'/exp OR 'videorecording' OR 'video' OR 'video'/exp OR video OR 'film' OR 'film'/exp OR film OR 'medical encounter' OR audiovisual OR webcam AND [embase]/lim OR ‘analogue-patient’

**Psychinfo (Advanced search. Exclude: ‘map term to subject heading’):**

(analogue patient*.mp. or lay people*.mp. or actor patient*.mp. or simulated patient*.mp. or placebo patient*.mp. or standardized patient*.mp. or lay person*.mp. or healthy patient*.mp. or healthy person*.mp. or healthy subject*.mp. or healthy participant*.mp. or naive patient*.mp. or lay subject*.mp. or naive participant*.mp. or naive subject*.mp.) AND (videotapes/ OR video display units/ OR audiovisual communications media/ OR video.mp. OR videotaped encounter OR film.mp. OR audiovisual.mp. OR webcam.mp.) OR analogue-patient

**CINAHL (Excluding Medline. Include: ‘also search in fulltext’)**

**Up to Februari 2010:**

(patient simulation(MH) OR analogue patient* OR actor patient* OR lay people* OR simulated patient* OR placebo patient* OR standardized patient* OR lay person* OR healthy patient* OR healthy person* OR healthy subject* OR healthy participant* OR naïve patient* OR lay subject* OR naïve participant* OR naïve subject*) AND (videorecording+(MH) OR audiovisual(MH) OR video OR film OR audiovisual OR webcam OR medical encounter) OR analogue-patient*

**From Februari 2010 to February 2012:**

(((MH "Patient Simulation") OR TX(analogue patient* OR actor patient* OR lay people* OR simulated patient* OR placebo patient* OR standardized patient* OR lay person* OR healthy patient* OR healthy person* OR healthy subject* OR healthy participant* OR naïve patient* OR lay subject* OR naïve participant* OR naïve subject*)) AND ((MH "Videorecording+") OR (MH "Audiovisuals+") OR TX(video OR film OR audiovisual OR web cam OR medical encounter))) OR TX(analogue-patient*)
